# Gene expression profiling of epithelium-associated FcRL4^+^ B cells in primary Sjögren’s syndrome reveals a pathogenic signature

**DOI:** 10.1016/j.jaut.2020.102439

**Published:** 2020-03-20

**Authors:** Gwenny M. Verstappen, John A. Ice, Hendrika Bootsma, Sarah Pringle, Erlin A. Haacke, Kim de Lange, Gerben B. van der Vries, Peter Hickey, Arjan Vissink, Frederik K.L. Spijkervet, Christopher J. Lessard, Frans G.M. Kroese

**Affiliations:** a Department of Rheumatology and Clinical Immunology, University of Groningen, University Medical Center Groningen, the Netherlands; b Genes and Human Disease Research Program, Oklahoma Medical Research Foundation, Oklahoma City, OK, USA; c Department of Pathology and Medical Biology, University of Groningen, University Medical Center Groningen, the Netherlands; d Department of Genetics, University of Groningen, University Medical Center Groningen, Groningen, the Netherlands; e Genomics Coordination Center, University of Groningen, University Medical Center Groningen, Groningen, the Netherlands; f The Walter and Eliza Hall Institute of Medical Research, 1G Royal Parade, Parkville, Melbourne, VIC, 3052, Australia; g Department of Medical Biology, University of Melbourne, Melbourne, VIC, 3010, Australia; h Department of Oral and Maxillofacial Surgery, University of Groningen, University Medical Center Groningen, the Netherlands; i Department of Pathology, University of Oklahoma Health Sciences Center, Oklahoma City, OK, USA

**Keywords:** Sjögren’s syndrome, B lymphocytes, Epithelium, Salivary gland, RNA sequencing, MALT lymphoma, Autoimmunity

## Abstract

In primary Sjögren’s syndrome (pSS), FcRL4^+^ B cells are present in inflamed salivary gland tissue, within or in close proximity to ductal epithelium. FcRL4 is also expressed by nearly all pSS-related mucosa-associated lymphoid tissue (MALT) B cell lymphomas, linking FcRL4 expression to lymphomagenesis. Whether glandular FcRL4^+^ B cells are pathogenic, how these cells originate, and how they functionally differ from FcRL4^−^ B cells in pSS is unclear. This study aimed to investigate the phenotype and function of FcRL4^+^ B cells in the periphery and parotid gland tissue of patients with pSS. First, circulating FcRL4^+^ B cells from 44 pSS and 54 non–SS–sicca patients were analyzed by flow cytometry. Additionally, RNA sequencing of FcRL4^+^ B cells sorted from parotid gland cell suspensions of 6 pSS patients was performed. B cells were sorted from cell suspensions as mini bulk (5 cells/well) based on the following definitions: CD19^+^CD27^−^FcRL4^−^ (‘naive’), CD19^+^CD27^+^FcRL4^−^ (‘memory’), and CD19^+^FcRL4^+^ B cells. We found that, although FcRL4^+^ B cells were not enriched in blood in pSS compared with non-SS sicca patients, these cells generally exhibited a pro-inflammatory phenotype. Genes coding for CD11c (*ITGAX*), T-bet (*TBX21*), TACI (*TNFRSF13B*), Src tyrosine kinases and NF-κB pathway-related genes were, among others, significantly upregulated in glandular FcRL4^+^ B cells versus FcRL4^−^ B cells. Pathway analysis showed upregulation of B cell activation, cell cycle and metabolic pathways. Thus, FcRL4^+^ B cells in pSS exhibit many characteristics of chronically activated, pro-inflammatory B cells and their gene expression profile suggests increased risk of lymphomagenesis. We postulate that these cells contribute significantly to the epithelial damage seen in the glandular tissue and that FcRL4^+^ B cells are an important treatment target in pSS.

## 1. Introduction

Primary Sjögren’s syndrome (pSS) is a systemic autoimmune disease that affects about 0.04–0.1% of the general population [1,2], and predominantly woman. Salivary and lacrimal glands are the main target tissue of the disease. Patients usually present with symptoms of dry mouth, dry eyes, and fatigue. Mononuclear infiltration, mainly consisting of CD4^+^ T cells and B cells, is a characteristic histopathological finding in pSS patients. Infiltrates are mostly concentrated around the ductal epithelium, but lymphocytes are also present within the epithelium where they may contribute to the formation of lymphoepithelial lesions (LELs) [3].

LELs are composed of proliferative metaplastic epithelial cells and intraepithelial lymphocytes. More severe LELs harbor larger numbers of intraepithelial B cells [4]. The vast majority of these cells express the inhibitory IgA-binding Fc receptor-like protein 4 (FcRL4) [5,6]. In healthy individuals, FcRL4^+^ B cells are restricted to mucosal tissues and mesenteric lymph nodes, where they participate in mucosal immune responses [7]. Interestingly, FcRL4^+^ B cells have also been found in the synovium of patients with rheumatoid arthritis (RA), where they produce high levels of RANKL and thereby exhibit a pathogenic role in this disease [8,9]. Analysis of immunoglobulin genes from FcRL4^+^ B cells in RA showed high levels of somatic hypermutation in the variable regions, which indicates that these cells are post-germinal center memory cells. The ratio of replacement mutations in FcRL4^+^ B cells indicated antigen-driven formation [8]. These studies in RA demonstrate that FcRL4^+^ B cells are activated cells that may play a major role in local immune responses in autoimmune diseases. We hypothesize that also in pSS, glandular FcRL4^+^ B cells contribute to pathogenesis by expressing pro-inflammatory factors and, as such, participate in LEL formation.

FcRL4^+^ B cells may also play a role in the development of mucosa-associated lymphoid tissue (MALT) lymphoma. This type of non-Hodgkin lymphoma is significantly more prevalent in pSS patients compared with population controls [10]. The neoplastic B cells of parotid MALT lymphomas are located in and around LELs, and widely express FcRL4 [5,11]. Notably, MALT lymphomas in pSS develop preferentially in the parotid glands, in which FcRL4^+^ B cells are more abundant compared to labial salivary glands [5].

Whether glandular FcRL4^+^ B cells are activated at extraglandular (mucosal) sites and subsequently migrate to the inflamed salivary glands of pSS patients, or whether they are formed locally in these glands is not known. We hypothesized that in a scenario of extraglandular formation and migration, the frequency of circulating FcRL4^+^ B cells would differ between pSS patients and non-SS sicca patients. To address this, we studied the frequency and phenotype of FcRL4^+^ B cells in the periphery using flow cytometry. To investigate how glandular FcRL4^+^ B cells contribute to pathogenesis, we sorted these cells from parotid glands of pSS patients and compared their gene expression profile with FcRL4^−^ B cells using mini-bulk RNA sequencing. Changes in gene expression between FcRL4^+^ and FcRL4^−^ B cells were used in pathway analyses to determine what, if any role, FcRL4^+^ B cells have in inflammation, LEL formation, and lymphomagenesis. We found that circulating FcRL4^+^ B cells have a pro-inflammatory phenotype, but frequencies were similar in pSS patients and non-SS sicca controls. Glandular FcRL4^+^ B cells had higher expression levels of genes involved in cell trafficking, B cell activation, and the NF-kappa B (NF-κB) pathway, linking these cells to LEL formation and, possibly, predisposition to lymphomagenesis.

## 2. Patients and methods

### 2.1. Immunophenotyping of circulating FcRL4^+^ B cells

Consecutive patients, referred to the University Medical Center Groningen (UMCG) for suspicion of SS were included in an inception cohort (*n* = 98). Informed consent was obtained according to the Declaration of Helsinki and the study was approved by the Medical Research Ethics Committee of the UMCG (METc2013.066). Inclusion criteria were age ≥18 years and sicca complaints. Patients who fulfilled 2016 ACR-EULAR criteria for SS were classified as pSS patients. Non-SS sicca patients were patients who did not fulfill 2016 ACR-EULAR criteria for SS. Patients diagnosed with other autoimmune diseases, hepatitis C, and HIV patients were excluded. From the 98 patients included in our cohort, 44 patients were classified as pSS and 54 as non-SS sicca patients. Of the 44 pSS patients, 80% were naive for treatment with corticosteroids or disease-modifying anti-rheumatic drugs. Two pSS patients were diagnosed with MALT lymphoma. Cryopreserved peripheral blood mononuclear cells were thawed and the frequency and phenotype of FcRL4^+^ B-cells were assessed by flow cytometry. The antibodies used are listed in Supplementary Table 1. Fixable viability dye eF506 (eBioscience) was used for live/dead discrimination. Data were acquired on a FACS-LSRII flow cytometer (Becton Dickinson, USA) and analyzed using FlowJo software (Tree Star, USA).

### 2.2. Tissue samples for RNA sequencing

FcRL4^+^ B cells are present in inflamed salivary gland tissue of patients with pSS, particularly in parotid gland tissue, but these cells are almost absent from salivary gland tissue of non-SS sicca patients and healthy individuals [5]. To investigate the phenotype and function of glandular FcRL4^+^ B cells in pSS patients, fresh parotid gland tissue was obtained from 6 adult patients who underwent a diagnostic biopsy. Patients were selected based on anti-SSA/Ro positivity and a high clinical suspicion of pSS. All patients fulfilled 2016 ACR-EULAR criteria for pSS. Surgeries were performed at the department of Oral and Maxillofacial Surgery of the UMCG. Permission to collect these tissues for research purposes was obtained from the Medical Research Ethics Committee of the UMCG (METc2016.010). Cell suspensions were prepared as described by Pringle et al. [12], with the following adaptions: biopsies were manually cut using scissors, the incubation period for enzyme-based digestion was 30 min and 32.5 μL digestion buffer was used per milligram of tissue.

### 2.3. Fluorescence-activated cell sorting for RNA sequencing

Fresh parotid gland cell suspensions were incubated with antibodies (identified below) for 30 min at 4 °C and washed twice in PBS/0.5% BSA/2 mM EDTA. The following antibodies were used: anti-human-CD19-eF450 (clone HIB19), anti-human-CD27-APC (clone O323), both from eBioscience, and anti-human-FcRL4-PE (clone 413D12, Biolegend). Immediately before sorting, cells were stained with propidium iodide (eBioscience) for live/dead discrimination. Gating was performed as described in Supplementary Fig. 1. Cells were sorted as 5 cells/well into 96-wells PCR plates containing 2 μl of lysis buffer (0.2% Triton X-100 (Sigma-Aldrich) + 2 U/μL RNAse inhibitor (Westburg-Clontech)), 1 μl of 10 μM oligo-dT_30_VN primer (Biolegio) and 1 μl of 4 × 10 mM dNTP mix (Westburg-Fermentas) per well. Cells were sorted on a MoFlo Astrios cell sorter (Beckman Coulter).

### 2.4. Preparation of cDNA libraries and sequencing

Complementary DNA (cDNA) library preparation was based on the Smart-seq2 protocol by Picelli et al. [13], but the following protocol adaptions were made to enable 3′-paired-end sequencing to decode cell barcodes and unique molecular identifiers (UMIs) from read 1: After a 3-min incubation–ligation step at 72 °C, a template switching oligo primer containing UMIs was bound to the poly-A tail of RNA transcripts, after which these were reverse transcribed using a reverse transcriptase (RT) mastermix (2.5 U SmartScribe RT, 0.25 U RNAse inhibitor (both from Westburg-Clontech), 1× SmartScribe first-strand buffer, 2 nM dithiothreitol (both from LifeTechnologies), 1 M betaine (BioUltra ≥99.0%; Sigma-Aldrich), 1 μM barcode-template switching oligo (BC-TSO; Biolegio)). After RT, an exonuclease step was added to remove unbound oligo-dT primers. One μL of exonuclease I (1:400 dilution in pure water) was added to each well and the plate was incubated 45 min at 37 °C, to activate the enzyme, immediately followed by 15 min at 85 °C to inactivate the enzyme. Pre-amplification of cDNA was performed using the KAPA HiFi HotStart ReadyMix (KAPA Biosystems) and BC-specific primers (fit at the end of the adapters). Samples were purified using Agencourt Ampure XP Beads (Beckman Coulter). The presence and size distribution of the obtained PCR product was measured on a PerkinElmer LabChip GX high-sensitivity DNA chip. Next, equimolar pooling of PCR products was performed. To tag the DNA with adapter sequences, a tagmentation step was incorporated using the Illumina Nextera XT DNA sample preparation kit, according to the manufacturer’s protocol, with 500 pg of pooled cDNA. Subsequently, the subpools were indexed using a N7xx primer from the Nextera XT DNA sample preparation kit and a custom P5-TSO hybrid primer (10 μM) with the Nextera PCR mastermix. The concentration and size distribution of the obtained Nextera products were measured on a PerkinElmer LabChip GX high-sensitivity DNA chip and a superpool was prepared by equimolar pooling of the six Nextera products. The superpool was divided over four lanes and sequenced on an Illumina NextSeq 500 instrument. The first read consisted of 17 bp, to sequence the cell-barcode (10 bp) and UMI (7 bp), followed by the index read sequencing the Nextera index (8 bp). The second read consisted of a 50 bp fragment, sequencing the last part of the captured gene. For mini-bulk samples, the median sequencing depth was 2.1*10^6^ reads per sample.

### 2.5. Read alignment, quality control and gene expression estimation

Dropseq tools 1.12 was used to extract well barcodes and UMIs from the reads [14]. The extracted well and UMI reads were flagged with bam tags and then stored in bam format together with the corresponding read. For quality control, only reads with a well and UMI barcode with a minimum basecallQuality of 10 were included. Of the remaining reads, the Smart adapter and PolyA tails were removed using Dropseq. In the next step, reads were aligned to the human genome (hg19) using STAR v2.5.1b with default settings [15]. The aligned reads were filtered for uniquely mapping reads. Before gene quantification, aligned reads were sorted using Picard tools 2.2.2 (https://broadinstitute.github.io/picard). Ensembl version 75 was used to map protein-coding transcripts. Quantification of gene expression was performed using Dropseq filtering on unique UMIs.

### 2.6. RNA expression data analysis

All statistical analysis and plotting of RNA-seq data was performed using R software v3.6.1 [16]. UMI-corrected read counts were used as input, correcting for PCR duplicates [17]. Immunoglobulin variable region gene transcripts were removed, because these sequences are not adjacent to the 3′ end and therefore, variance in amplification of the entire variable region is expected between samples with the current method. For differential expression analysis of mini-bulk samples, DESeq2 v1.24.0 was used [18], following the recommendations for single-cell data, because of the high amount of zero’s in the dataset. Six (biological) replicates per cell subset per patient were included in the analysis. Genes with ≤1 read across all samples were first removed from the dataset. The DESeq2 model included the cell type (condition) and patient number (batch). Approximate posterior estimation for generalized linear model (apeglm) was used to estimate the logarithmic fold change [19]. Differentially expressed genes were analyzed for pathway enrichment using Ingenuity Pathway analysis (QIAGEN Inc., USA) [20].

### 2.7. Immunohistochemistry

Formalin-fixed (4%), paraffin-embedded parotid gland tissue sections (4 μm) of five pSS patients from the inception cohort were included. Patients were selected based on the presence of LELs (identified by H&E staining). After deparaffinization and heat-induced antigen retrieval in sodium citrate buffer (pH 6.0) for 20 min and endogenous peroxidase (0.3% H2O2) blocking, Ultra V Block (ThermoFisher Scientific) was applied for 5 min. Slides were incubated overnight at 4 °C with 2.5 μg/mL monoclonal mouse anti-human PAX5 antibody (BD Biosciences, clone 24/Pax-5) and 1 μg/mL monoclonal rabbit antihuman CD11c antibody (Abcam, clone EP1347Y) diluted in PBS/1% BSA/0.05% Tween-20. Secondary antibody-based detection was performed using the MultiVision polymer detection system (anti-rabbit/HRP and anti-mouse/AP polymers; ThermoFisher Scientific) according to the manufacturer’s instructions.

### 2.8. Statistical analysis

The Mann-Whitney *U* test was used to compare frequencies and numbers of FcRL4^+^ B cells in blood between pSS patients and non-SS sicca patients. P-values < 0.05 were considered statistically significant. For statistical analysis of mini-bulk RNA sequencing, we used DESeq2 software with a likelihood ratio test design [18]. P-values for differentially expressed genes were adjusted by the Benjamini-Hochberg method and are hereafter indicated as false detection rate (FDR).

## 3. Results

### 3.1. Immunophenotyping of circulating FcRL4^+^ B cells in pSS and non-SS sicca patients

**Fig. 1.**
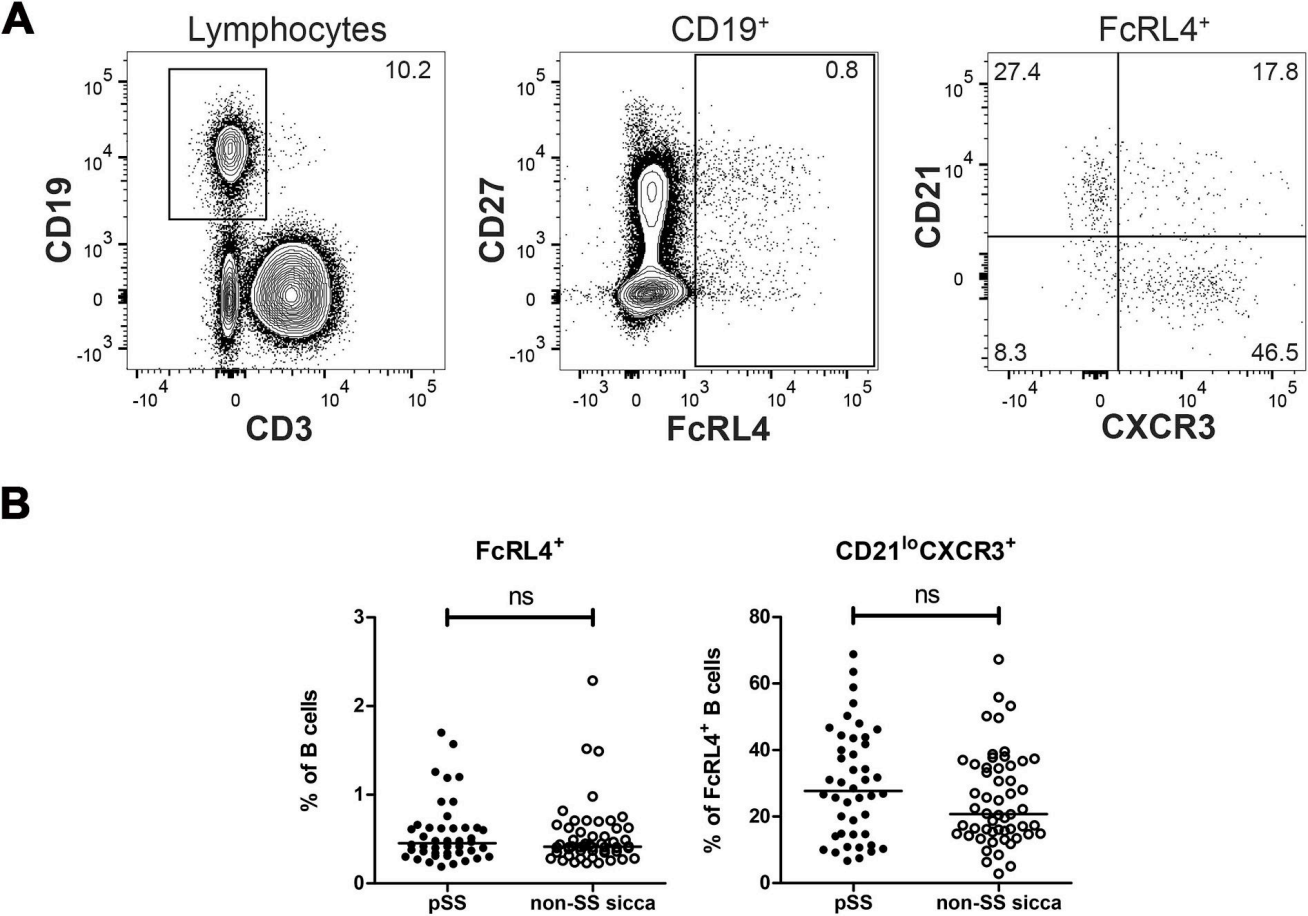
FcRL4^+^ B cells in peripheral blood of pSS patients and non-SS sicca patients. (A) Gating strategy used to identify FcRL4^+^ B cells in peripheral blood. Lymphocytes were gated from single, live cells using forward and side scatter properties and fixable viability dye staining. (B) Frequencies of FcRL4^+^ cells within the B-cell compartment and frequencies of CD21^lo^CXCR3^+^ cells within the FcRL4^+^ B-cell compartment are shown. Data from pSS patients (*n* = 44) and non-SS sicca patients (*n* = 54) were included. P-value < 0.05 was considered significant. Mann-Whitney *U* test was used for statistical analysis. Ns = not significant.

First, we compared frequencies of circulating FcRL4^+^ B cells between pSS and non-SS sicca patients. Patient characteristics are summarized in Table 1. We measured co-expression of multiple surface markers expressed by B cells, including CD27, CD21, and CXCR3 (Fig. 1A). We found no significant differences in frequencies of circulating FcRL4^+^ B cells between pSS and non-SS sicca patients (Fig. 1B). Also, absolute numbers of these cell subsets were not significantly altered (data not shown). Two pSS patients with MALT lymphoma did not show aberrant frequencies of these cells either. Circulating FcRL4^+^ B cells comprised both CD27^−^ and CD27^+^ cells. A large proportion of circulating FcRL4^+^ B cells expressed low levels of CD21 and co-expressed CXCR3 (Fig. 1B). We did not observe significant phenotypical differences in circulating FcRL4^+^ B cells between pSS and non-SS sicca patients. Their low prevalence notwithstanding, CXCR3 expression of circulating FcRL4^+^ B cells suggests that these cells do have the capacity to migrate to inflamed tissue sites.

### 3.2. RNA sequencing of B cells isolated from parotid glands of pSS patients

**Fig. 2.**
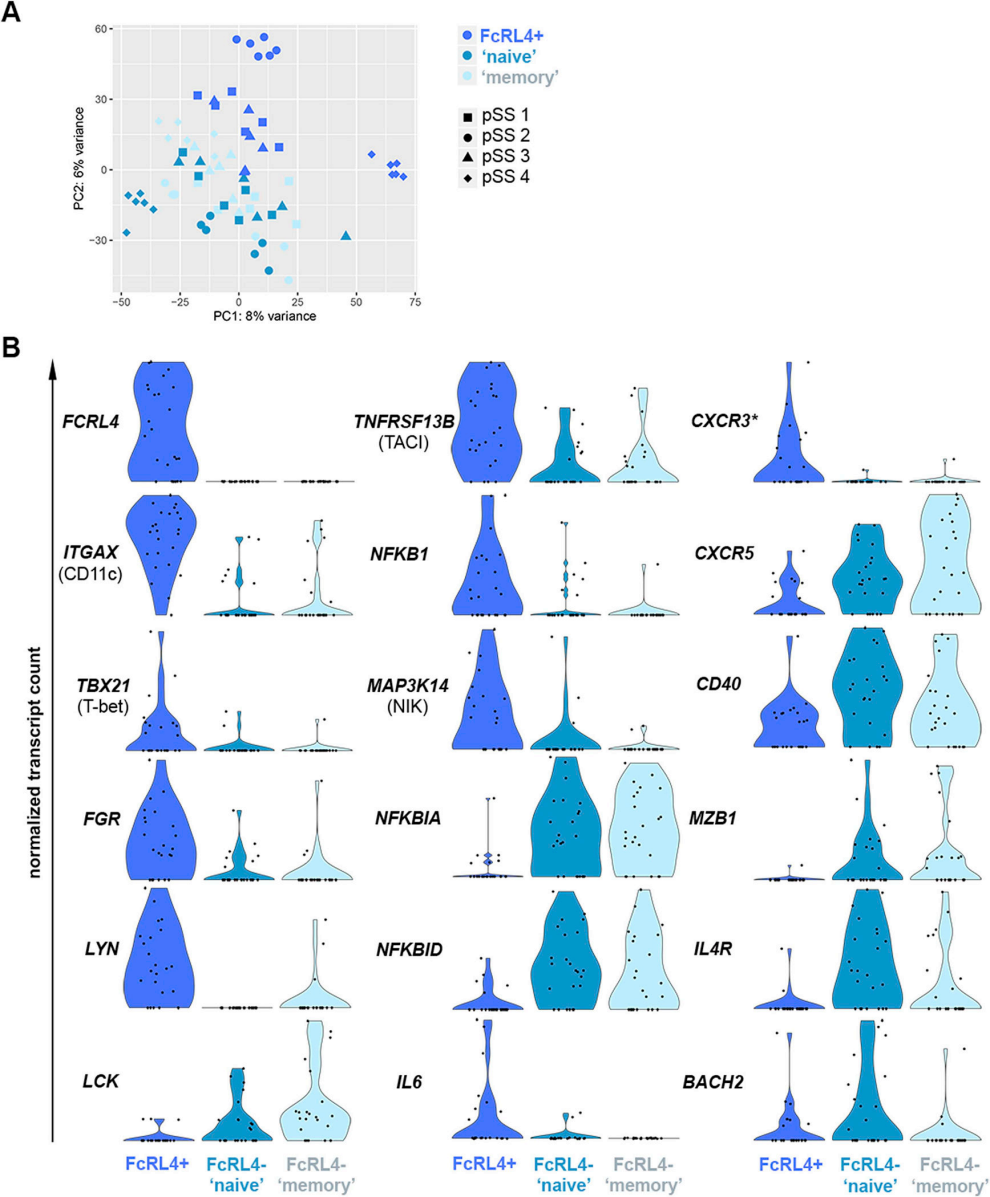
Differential gene expression in FcRL4^+^ B cells. Clustering and differential gene expression for mini-bulk samples were analyzed using DESeq2 software. (A) Principal Component Analysis (PCA) plot of all included samples, colored by (flow cytometry-based) cell subset. (B) Violin plots showing normalized transcript counts (y-axis) per cell type (x-axis) for genes with known immune function that were differentially expressed in FcRL4^+^ B cells, compared with both FcRL4^−^ subsets (FDR < 0.05). Each black dot represents a 5-cell sample. * Not marked as differentially expressed gene in statistical analysis.

Also in the inflamed salivary gland tissue of patients with pSS, the number of FcRL4^+^ B cells is relatively low, albeit higher in parotid compared to labial glands [5]. Here, FcRL4^+^ B cells concentrate around the ductal epithelium [5]. In contrast to pSS, B cells are nearly absent around and within the striated ducts in salivary gland tissue of non-SS sicca patients [4]. To begin exploring the potential role of FcRL4^+^ B cells, we sorted these cells, as well as FcRL4^−^ B cell subsets, from parotid gland tissue of six patients with pSS and then performed RNA sequencing. Gene expression profiles were compared between FcRL4^+^, FcRL4^−^CD27^−^ ‘naive’ and FcRL4^−^CD27^+^ ‘memory’ B cells. One patient sample (out of six) was excluded because of low RNA yields during library preparation. Of the remaining five patients, one was diagnosed with MALT lymphoma. Data from this patient were excluded from differential expression analysis, because the transcriptional profile of these cells may bias the analysis of non-lymphoma B cells. Characteristics of the patients included (n = 4) are shown in Table 2. Data from mini-bulk samples (5 cells per subset per patient, 6 replicates per subset) from four patients were used to compare the gene expression profiles between B cell subsets. Principal component (PC) analysis did not show very distinct clustering of the sorted subsets, although FcRL4^+^ cells from three patients clustered separately in PC2 (Fig. 2A). By using DESeq2 software to compare FcRL4^+^ B cells with FcRL4^−^CD27^−^ ‘naive’ B cells and FcRL4^−^CD27^+^ ‘memory’ B cells, we identified 526 differentially expressed genes in FcRL4^+^ B cells (DEG; FDR < 0.05).

Next, we evaluated differentially expressed genes with a focus on genes with known immune function (Fig. 2B). We found differential expression of several genes previously associated with FcRL4^+^ B cells [8,21,22]. These genes included *ITGAX* (CD11c), *TBX21* (T-bet), *CXCR5, CD40* and the Src tyrosine kinases *FGR, LYN,* and *LCK*. Although *CXCR3* was not marked as a DEG, we did observe upregulation of *CXCR3* in FcRL4^+^ B cells (Fig. 2B). At the same time, expression of lymphoid tissue homing factors *SELL* (CD62L) and *CCR7* was reduced (Supplementary Table 2). Upregulation of CD11c and CXCR3, and simultaneous downregulation of CXCR5, CD62L, and CCR7 are important for cell trafficking into inflamed tissues. We also found upregulation of genes associated with activation of both the canonical and non-canonical NF-κB signaling pathway: *TNFRSF13B* (TACI), *NFKB1* (p50), and *MAP3K14* (NIK). Expression levels of *NFKBIA* (IκBα) and *NFKBID* (IκBNS), negative regulators of NF-κB, were significantly downregulated in FcRL4^+^ B cells. Although we did not detect many transcripts coding for cytokines, *IL6* was significantly upregulated. Genes that are involved in regulation of B cell differentiation, i.e. *MZB1*, *IL4R*, and *BACH2*, were expressed at lower levels in FcRL4^+^ B cells (Fig. 2B). Other possibly relevant genes upregulated in these cells were *CCR5*, *CD97* and *ITGA4* (cell trafficking) as well as *IFNGR1* and *IL27RA* (interferon type II-mediated response). A full list of differentially expressed genes with FDR < 0.1 is included as Supplementary Table 2.

### 3.3. Pathway analysis of differentially expressed genes

**Fig. 3.**
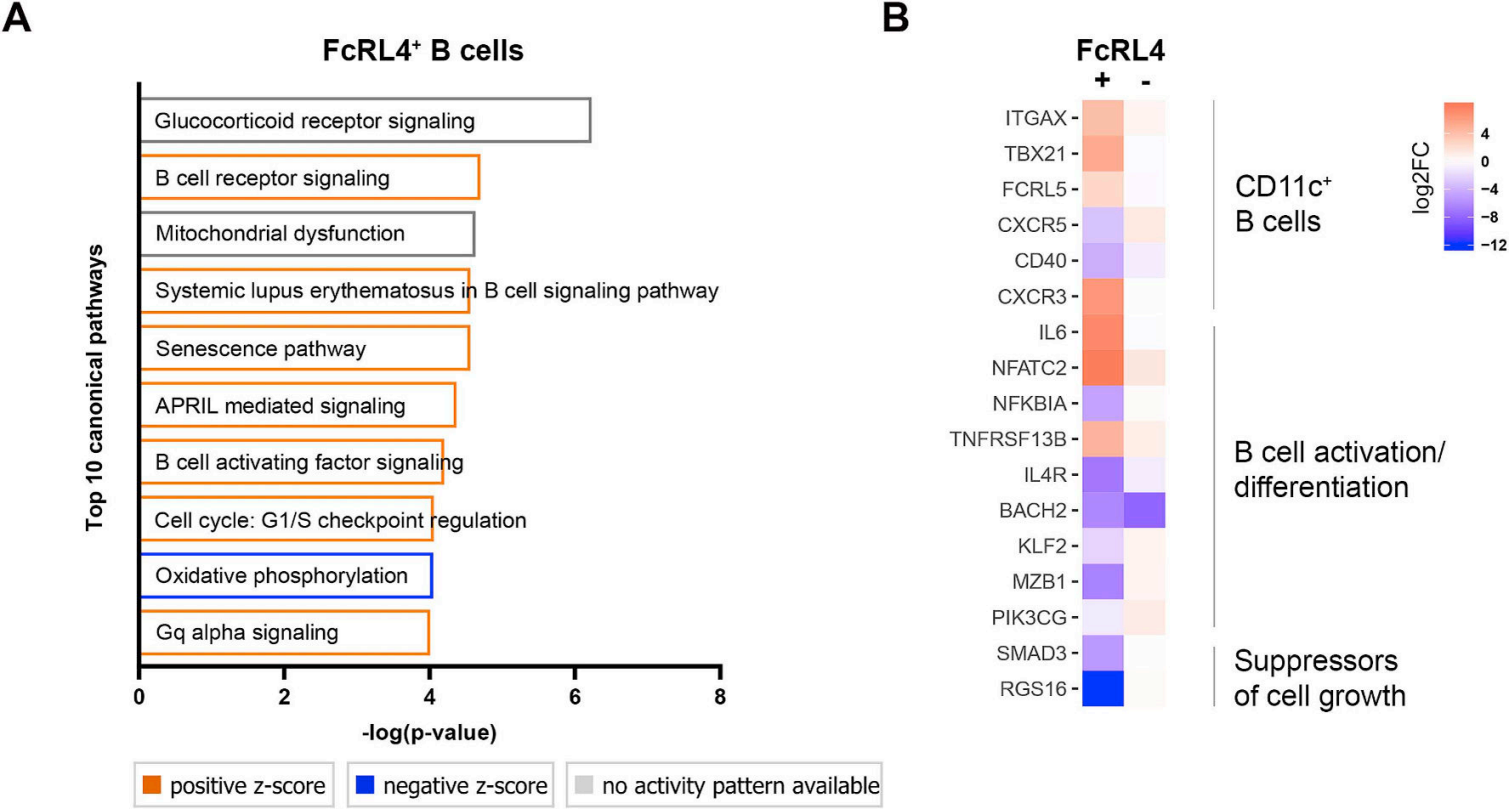
Pathway analysis in FcRL4^+^ B cells. (A) Pathway enrichment was assessed using Ingenuity Pathway Analysis (IPA). A list of differentially expressed genes (DEG) between FcRL4^+^ B cells and FcRL4^−^CD27^+^ ‘memory’ B cells, with an FDR < 0.1, was used as input. Pathways related to B cell activation and cell cycle were, amongst others, significantly enriched in FcRL4^+^ B cells. Z-scores are a measure of the match between expected relationship direction and observed gene expression. (B) Specific patterns of gene expression in FcRL4^+^ B cells are presented. Colors indicate shrunken log 2 fold change values of DEG genes with FDR < 0.05. (For interpretation of the references to color in this figure legend, the reader is referred to the Web version of this article.)

Using Ingenuity Pathway Analysis (IPA), we found many differentially regulated canonical pathways in FcRL4^+^ B cells, compared with FcRL4^−^ ‘memory’ B cells (Supplementary Table 3). Pathways involved in B cell activation, metabolic and cell cycle-related pathways were particularly enriched in FcRL4^+^ B cells (Fig. 3A). In addition to IPA, we could identify specific patterns of gene expression associated with CD11c^+^ B cells [23], B cell differentiation, and tumor suppression in FcRL4^+^ B cells (Fig. 3B).

### 3.4. Presence of CD11c^+^ B cells in parotid gland tissue

**Fig. 4.**
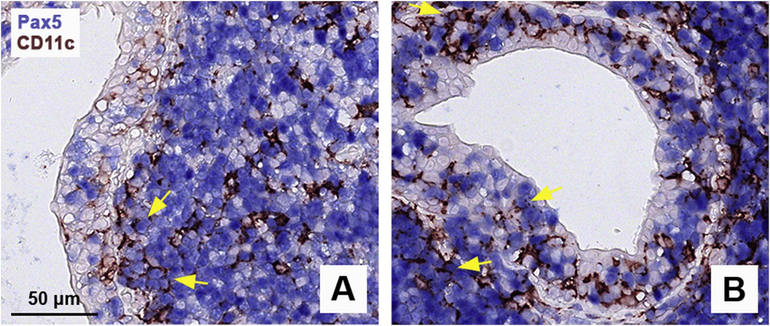
Presence of CD11c^+^ B cells in parotid gland tissue of pSS patients. Double staining for CD11c (brown) and the B-cell lineage-specific transcription factor Pax5 (blue) in two pSS patients with lymphoepithelial lesions (LELs) (A–B). Large numbers of B cells are present within LELs and around the inflamed ducts. Single CD11c^+^ positive cells indicate dendritic cells. Examples of CD11c^+^Pax5^+^ cells are indicated by yellow arrows. (For interpretation of the references to color in this figure legend, the reader is referred to the Web version of this article.)

Since *ITGAX* (CD11c) was significantly upregulated in FcRL4^+^ B cells, compared with FcRL4^−^ B cells, we aimed to confirm the presence of CD11c^+^ B cells in inflamed parotid gland tissue from pSS patients with LELs. Because CD11c is also expressed by dendritic cells, CD11c^+^ B cells were characterized by co-expression of CD11c and the B-cell lineage-specific transcription factor Pax5. CD11c^+^ B cells were indeed present in periductal infiltrates of pSS patients (Fig. 4A). However, within the ductal epithelium, only a fraction of B cells showed positivity for CD11c (Fig. 4B), while FcRL4 was shown to be expressed by the vast majority of these cells [5].

## 4. Discussion

FcRL4 is expressed by epithelium-associated B cells in the salivary glands of pSS patients [5]. However, the origin, phenotype and function of FcRL4^+^ B cells in pSS remain poorly understood. In addition to immunophenotyping of circulating FcRL4^+^ B cells, we investigated, for the first time, the gene expression profile of glandular FcRL4^+^ B cells that were isolated from parotid gland tissue of pSS patients. Glandular FcRL4^+^ B cells showed upregulation of multiple genes involved in B cell trafficking and B cell activation. Our results indicate that FcRL4^+^ B cells in pSS exhibit many characteristics of chronically activated, proinflammatory, CD11c^+^T-bet^+^ B cells, which were recently shown to be involved in the pathogenesis of systemic lupus erythematosus [24,25]. We postulate that FcRL4^+^ B cells contribute significantly to the epithelial damage seen in the glandular tissue of pSS patients.

Glandular FcRL4^+^ B cells were studied using mini-bulk RNA sequencing. We used cell sorting to enrich for FcRL4^+^ and FcRL4glandular B cells. This approach allowed us to partially overcome the limited number of cells and fresh biopsy samples available. Gene expression analysis of the mini-bulk samples showed upregulation of *CXCR3* almost exclusively in FcRL4^+^ B cells, together with downregulation of *CXCR5*. Similarly, flow cytometric analysis of circulating B cells showed that a large proportion of FcRL4^+^ B cells in blood coexpresses CXCR3. This may explain inflammation-induced homing to the ductal epithelial cells of the affected salivary glands, which secrete high levels of the chemokine CXCL10/IP-10 [26], the ligand for CXCR3. Additionally, integrins (e.g., *ITGAX* (CD11c)) and other adhesion molecules (e.g., *CD97*) were upregulated in FcRL4^+^ B cells, compared with FcRL4^−^ B cells. The expression of CD11c by B cells in periductal infiltrates and LELs within parotid gland tissue of pSS patients was confirmed by immunohistochemistry, albeit that CD11c was not expressed by all intraepithelial B cells. Since CD11c is considered to be a marker of recent activation [25,27], our results suggest that FcRL4^+^ B cells have been activated at the epithelial border. Subsequently, the expression of CD11c and other adhesion molecules by these cells may result in retention of these cells around and within the epithelium by interaction with their ligands, such as VCAM-1 and ICAM-1. FcRL4^+^ B cells further exhibited increased transcript expression of Src family kinases (*HCK*, *FGR*, *LYN*), which are important for integrin signal transduction [28]. The phenotype of glandular FcRL4^+^ B cells is thus consistent with results from earlier studies that analyzed the transcription profile of FcRL4^+^ B cells in tonsils, the blood of HIV-viremic individuals or synovia of patients with RA [8,21,22].

Previous studies have shown that enhanced expression of CD11c by memory B cells is associated with multiple autoimmune conditions and chronic immune stimulation [23]. CD11c^+^ memory cells are atypical memory cells, characterized by low expression of CD27 and low expression of CD21. These cells exhibit autoreactive specificities, are refractory to BCR stimulation, and respond robustly to TLR activation [23]. A similar pattern of downregulated BCR signaling and enhanced TLR signaling has been shown for FcRL4^+^ B cells [29]. There is some evidence that binding of IgA to FcRL4 on the B cells is important for this switch from adaptive to innate signaling [29]. Negative regulation of BCR-induced signaling may be established by upregulation of *LYN,* as we observed in glandular FcRL4^+^ B cells. Lyn is a Src tyrosine kinase that can initiate, but mostly negatively regulates BCR signaling [30]. The transcriptional profile of FcRL4^+^ B cells from parotid glands further indicates that these cells have indeed been activated, as upregulation of multiple genes involved in NF-κB signaling was seen. One of these genes was *TNFRSF13B*, coding for TACI, a receptor that binds both BAFF and APRIL. Upon binding, NF-κB signaling and B cell survival are induced [31]. BAFF and APRIL are significantly upregulated in the salivary glands of pSS patients and are also produced by the ductal epithelial cells [32]. Thus, binding of BAFF and/or APRIL to TACI expressed by FcRL4^+^ B cells may promote their activation and survival. Unexpectedly, the upregulation of NF-κB pathway genes in glandular FcRL4^+^ B cells was not accompanied by increased expression levels of genes encoding for pro-inflammatory cytokines, except for *IL6*, which was significantly upregulated in some FcRL4^+^ B cells. Low abundance of cytokine transcript detection is a known issue in (single-cell) RNA sequencing and may be due to short half-life of cytokine mRNAs. Thus, additional effector functions of FcRL4^+^ B cells that may contribute to epithelial damage and formation of LELs need further investigation.

In contrast to findings regarding CD11c^+^T-bet^+^ B cells in SLE, we did not find evidence that FcRL4^+^ B cells are differentiating into antibody-secreting cells (ASC), as the required transcription factors (e.g. *IRF4*, *PRDM1*, *XBP1* [33]) were either downregulated or not detected. This is in line with our previous histologic observation that FcRL4^+^ B cells lack IRF4 and Blimp-1 protein expression [5]. *MZB1*, which is important for differentiation of marginal zone B cells into ASC [34], was also downregulated. Oppositely, the expression of *BACH2*, which is a negative regulator of effector B cell differentiation and highly expressed by naïve B cells, was reduced in FcRL4^+^ B cells, similar to what has been observed in ASC [35]. Together our results suggest that, despite their activated state, the expression of FcRL4^+^ puts a brake on ASC formation.

FcRL4 is not only expressed by epithelium-associated B cells, but also by MALT lymphomas [11]. The clear co-localization of neoplastic B cells with epithelial cells in MALT lymphomas suggests that this disease depends on the interaction between B cells and epithelial cells. We therefore speculate that the highly proliferative, activated FcRL4^+^ B cells may become neoplastic B cells [5]. In support of this notion, we observed upregulation of several genes in FcRL4^+^ B cells that are associated with lymphomagenesis. Firstly, expression levels of several NFκB pathway genes are increased in FcRL4^+^ B cells as well as in MALT lymphoma and the vast majority of MALT lymphoma-associated gene translocations is associated with NF-κB activation [36]. In pSS, a germline missense polymorphism in *TNFAIP3*, a gene coding for an important negative feedback regulator of the NF-κB pathway, is associated with lymphoma development, particularly in patients with early disease onset [37,38]. Also, NF-κB2 was upregulated in B cells from SS patients with lymphoma who harbored a missense mutation in the *TNFRSF13C* (BAFF-R) gene [39]. These findings underline that impaired control of NF-κB activation increases the risk of lymphoma. An additional factor that may contribute to lymphomagenesis is upregulation of genes that promote cell survival, such as *TNFRSF13B* [40], and downregulation of pro-apoptotic and tumor suppressor genes (*SMAD3* [41]*, RGS16* [42,43]) in FcRL4^+^ B cells, probably saving them from apoptosis. Finally, we found upregulation of *IL27RA*, which is specifically overexpressed in MALT lymphoma compared with other B-cell lymphomas [44]. In mice, IL-27 signals directly to B cells and promotes differentiation towards germinal center (GC) B cells via STAT1 [45]. IL27R was expressed on GC B cells following CD40 stimulation, inducing STAT1 phosphorylation in humans [46]. IL-27 is also able to induce T-bet expression in naive and memory B cells [46,47].

In addition to increased activity of the IL-27R/STAT1/T-bet axis in FcRL4^+^ B cells, observed in the current study, others have shown that expression levels of IFN-γ and type-II IFN inducible genes were increased in minor salivary gland tissue of SS patients with versus without lymphoma [48]. These data suggest that a broad type 1 immune response, coordinated by T-bet expression in different cell types, is associated with lymphomagenesis in pSS patients. A possible explanation for this association is that T-bet in B cells controls expression of activation-induced cytidine (AID) [49], which is essential for somatic hypermutation and isotype switching in B cells. A previous study showed that FcRL4^+^ memory B cells have higher expression levels of *AICDA* (coding for AID) than FcRL4^−^ memory B cells [21]. Importantly, AID can also target many other genes outside immunoglobulin loci, which may result in off-target, potentially oncogenic, mutations [50]. Although *AICDA* was not a differentially expressed gene in our dataset, low transcript counts of this gene were found in some FcRL4^+^ samples. We hypothesize that extra-follicular expression of *AICDA* in FcRL4^+^ B cells may contribute to transformation of these cells towards neoplastic MALT lymphoma cells.

A limitation of our study is the small number of patient samples, which was due to the low frequency of available fresh parotid gland biopsies from patients that fulfilled our inclusion criteria (high suspicion of pSS and anti-SSA positivity). Another limitation could be that the average age of the patients included is relatively high (68 years). We did not select patients by age nor by histopathological characteristics. Nonetheless, to our best knowledge this is the first study to reveal the gene expression profile of FcRL4^+^ B cells isolated from salivary gland tissue of pSS patients. FcRL4^+^ B cells show similarities in gene expression profile to chronically activated CD11c^+^T-bet^+^ memory B cells in systemic lupus erythematosus patients [24], as well as strong NF-κB activation. Specific chemokine receptor and integrin expression may be responsible for guidance to and cross-talk with epithelial cells that form LELs. By interacting with epithelial cells and secreting IL-6, FcRL4^+^ B cells may contribute to pSS histopathology and hyposalivation. Lastly, we show that FcRL4^+^ B cells isolated from glandular tissue of pSS patients express anti-apoptotic factors that, combined with a high proliferative capacity and possibly somatic hypermutation, may put them at risk of lymphomagenesis. Thus, B-cell targeting therapies, such as rituximab, that deplete FcRL4^+^ B cells and restore of the ductal epithelium may be beneficial in patients with pSS and even prevent MALT lymphoma development within the salivary glands.

## Supplementary Material

1

3

2

4

## Figures and Tables

**Table 1:** Baseline characteristics of the inception cohort used for immunophenotyping of FcRL4^+^ B cells in blood.

Characteristic	pSS (n = 44)	non-SS sicca (n = 54)
Age, median (IQR), years	54 (45–62)	48 (40–56)
Female gender, n (%)	42 (96)	46 (85)
Xerostomia, n (%)	42 (96)	52 (96)
Keratoconjunctivitis sicca, n (%)	39 (89)	52 (96)
UWSF ≤ 0.1 mL/min, n (%)	23 (52)	22 (41)
Schirmer’s test ≤ 5mm/5 min, n (%)	36 (82)	31 (57)
OSS ≥ 5, n (%)	19 (43)	6 (11)
Parotid gland enlargement, n (%)	18 (41)	8 (15)
ESSDAI, median (IQR)	4 (1–9)	–
IgG (g/L), median (IQR)	16 (12–20)	10 (8–12)
Anti-Ro/SSA positive, n (%)	33 (75)	3 (6)
Anti-La/SSB positive, n (%)	18 (41)	0 (0)
RF positive, n (%)	26 (59)	1 (2)

IQR: Interquartile range; UWSF: Unstimulated whole salivary flow; OSS: Ocular Staining Score; ESSDAI: EULAR Sjögren’s Syndrome Disease Activity Index; RF: Rheumatoid factor.

**Table 2:** Demographic, clinical and histologic characteristics of patients included for parotid gland B cell RNA sequencing.

	Age (years)	Gender	IgG (g/L)	Anti-Ro52 positive	Anti-Ro60 positive	Anti-La positive	IgM-RF (kIU/L)	Chisholm score	Focus score (per 4 mm^2^)	Presence of LEL	Presence of GC	Plasma cell shift^a^
Patient 1	74	F	8	Yes	No	No	8.8	NA^b^	NA^b^	+	−	+
Patient 2	69	F	25	Yes	Yes	No	39	4	1.1	−	−	+
Patient 3	61	F	20	Yes	Yes	Yes	11	4	3.2	+	−	+
Patient 4	68	F	27	Yes	Yes	Yes	33	4	1.2	+	−	+

RF: Rheumatoid factor; LEL: Lympho-epithelial lesion; GC: Germinal center; NA: Not assessed.

a > 30% IgG-containing plasma cells over < 70% IgA-containing plasma cell in diagnostic biopsy.

b Chisholm and focus score could not be calculated because of atrophy and a limited area of parenchyma in the diagnostic biopsy. In the parenchyma that was left, we were able to identify, however, LELs and a plasma cell shift towards IgG. These two findings are indicative of pSS.
