# Supplementary material for: Gene expression profiling of epithelium-associated FcRL4+ B cells in primary Sjögren’s syndrome reveals a pathogenic signature
Source: J Autoimmun. Author manuscript; Available in PMC 2020 Jul 6. (PMC7337041; doi:10.1016/j.jaut.2020.102439)
Supplement: 1 [file NIHMS1601970-supplement-1.docx]

**Supplementary Table 1.** Antibodies used for immunophenotyping of FcRL4^+^ B cells.

| **Antigen** | **Fluorochrome** | **Clone** | **Company** |
| --- | --- | --- | --- |
| CD19 | BV786 | SJ25C1 | BD Biosciences |
| CD27 | BV421 | M-T271 | BD Biosciences |
| IgD | BUV395 | IA6-2 | BD Biosciences |
| CD21 | BUV737 | B-ly4 | BD Biosciences |
| CXCR3 | PE-Cy7 | 1C6 | BD Biosciences |
| FcRL4 | PE | 413D12 | Biolegend |
