# Supplementary figures and images for: Gene expression profiling of epithelium-associated FcRL4+ B cells in primary Sjögren’s syndrome reveals a pathogenic signature
Source: J Autoimmun. Author manuscript; Available in PMC 2020 Jul 6. (PMC7337041; doi:10.1016/j.jaut.2020.102439)

**1. Parotid gland cells**

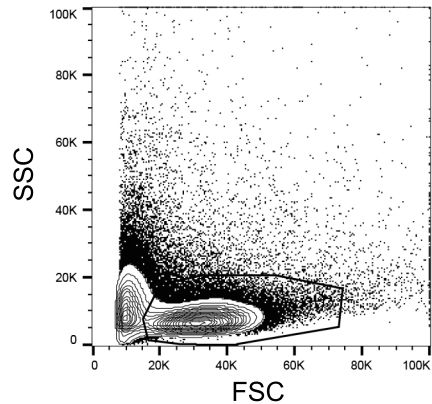

**2. Lymphocytes**

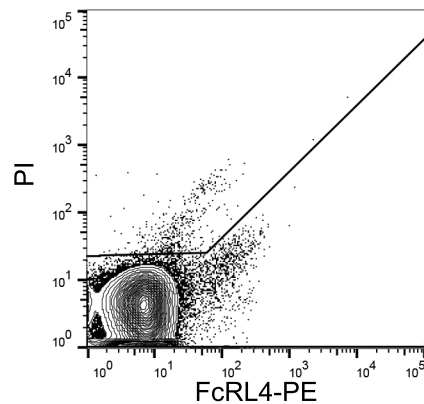

**3. Live lymphocytes**

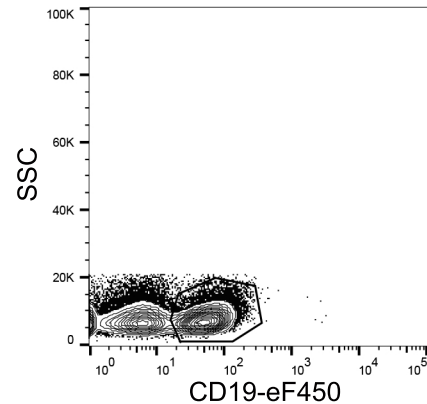

**4. B cells**

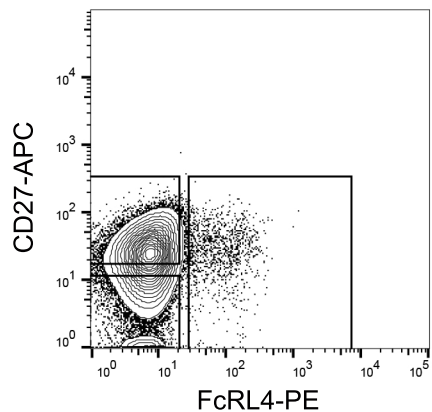

**B cells (PBMC control)**

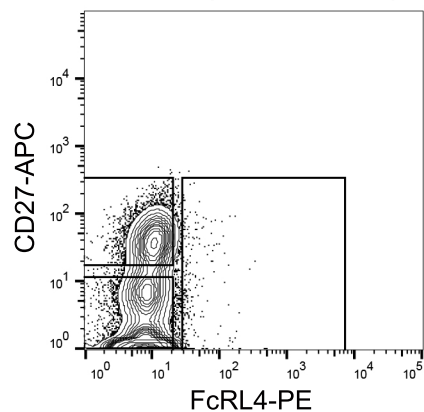

**FMO PE/APC**

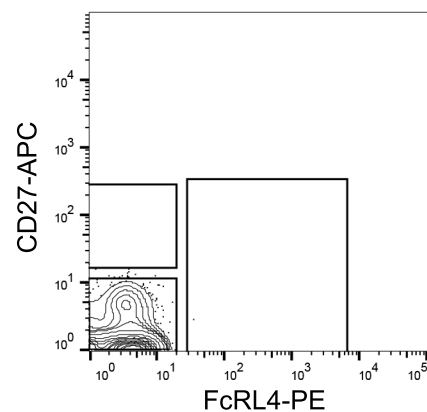

Supplement: 4 [file NIHMS1601970-supplement-4.pdf]
